# Recurrent takotsubo cardiomyopathy in the setting of transient neurological symptoms: a case report

**DOI:** 10.1186/1752-1947-5-412

**Published:** 2011-08-24

**Authors:** Muhammad Rizwan Sardar, Catherine Kuntz, Jeremy A Mazurek, Naveed Hassan Akhtar, Wajeeha Saeed, Timothy Shapiro

**Affiliations:** 1 Congestive Heart Failure Program, Division of Cardiology, Montefiore Medical Center, 1825 Eastchester Road, Suite W-195, Bronx, NY 10461, USA; 2 Division of Pulmonology and Critical Care, Lankenau Medical Center, Lankenau Hospital MOB West, Suite 230, 100 Lancaster Avenue, Wynnewood, PA 19096, USA; 3 Department of Medicine, Jacobi Medical Center, Albert Einstein College of Medicine, 1400 Pelham Parkway South, Suite 3N1, Bronx, NY 10461, USA; 4 Department of Medicine, Weill-Cornell Medical College, 1300 York Avenue, New York, NY 10065, USA; 5 Division of Cardiology, Lankenau Medical Center, Campus Chief and Program Director, Interventional Cardiology, Lankenau Medical Science Building, Suite 380, 100 Lancaster Avenue, Wynnewood, PA 19096, USA

## Abstract

**Introduction:**

First described in Japan, takotsubo cardiomyopathy is increasingly becoming recognized worldwide as a cause of sudden and reversible diminished left ventricular function characterized by left apical ballooning and hyperkinesis of the basal segments, often with symptoms mimicking a myocardial infarction. Associated with physical or emotional stress, its exact pathogenesis has not been established, though evidence supports a neurohumoral etiology. Additionally, recurrence of this condition is rare. In this report, we present a rare case of recurrent takotsubo cardiomyopathy in a post-menopausal woman who presented with transient neurological complaints on both occasions.

**Case presentation:**

We present a rare case of a 76-year-old Caucasian woman with no history of congestive heart failure who presented to our emergency department twice with transient neurological complaints. On the first occasion, she was found to have transient aphasia which resolved within 24 hours, yet during that period she also developed symptoms of congestive heart failure and was noted to have a new, significantly depressed ejection fraction with apical akinesis and possible apical thrombus. One month after her presentation a repeat echocardiogram revealed complete resolution of all wall motion abnormalities and a return to baseline status. Seven months later she presented with ataxia, was diagnosed with vertebrobasilar insufficiency, and again developed symptoms and echocardiography findings similar to those of her first presentation. Once again, at her one-month follow-up examination, all wall motion abnormalities had completely resolved and her ejection fraction had returned to normal.

**Conclusion:**

Though the exact etiology of takotsubo cardiomyopathy is unclear, a neurohumoral mechanism has been proposed. Recurrence of this disorder is rare, though it has been reported in patients with structural brain abnormalities. This report is the first to describe recurrent takotsubo cardiomyopathy in a patient with transient neurological symptoms. In our patient, as expected in patients with this condition, complete resolution of all left ventricular abnormalities occurred within a short period of time. It is important for clinicians to be aware of this increasingly recognized syndrome, including its association with recurrence, especially in the clinical setting of neurologic dysfunction.

## Introduction

Left apical ballooning syndrome, also known as takotsubo cardiomyopathy (TTC), is a clinical syndrome of transient diminished left ventricular (LV) apical wall motion with relative preservation of the basal heart segment in the presence of normal coronary arteries. It was first described in Japan in the early 1990s [1] and is named for ventricles which seem similar in appearance to a Japanese octopus trap on ventriculography scans. This syndrome has been linked to emotional or physical stress, with a high incidence among post-menopausal women [2]. However, the etiology of TTC is not completely understood. Several possible mechanisms, including microvascular dysfunction, coronary artery vasospasm, aborted myocardial infarction, and excess catecholamine stimulation, have been proposed [2,3]. Typically, LV function returns to normal within six to eight weeks. Recurrence, which is increasingly being reported in the literature [2,4,5], can be related to neurological pathology [6,7]. We present a case of a woman with recurrent TTC whose presenting symptoms on both occasions were of neurological origin.

## Case presentation

A 76-year-old Caucasian woman presented to our tertiary care hospital on two separate occasions. She had a medical history significant for hypertension, diabetes mellitus, dyslipidemia, hypothyroidism, no congestive heart failure (CHF), no evidence of coronary artery disease visualized by catheterization two years prior to her initial presentation, and a normal baseline left ventricular ejection fraction (LVEF) of 55% to 60%. In each episode, she presented without evidence of existing physical or emotional distress.

At her first presentation, she arrived at the emergency department with the onset of aphasia in the setting of hypertensive emergency (blood pressure (BP) 226/100 mmHg). Her aphasia was isolated and transient, and her physical examination was otherwise unremarkable. Because we were concerned about an acute intracerebral event in the setting of hypertensive emergency, computed tomography and diffusion-weighted MRI/magnetic resonance angiography (MRI/MRA) of the brain were performed, both of which showed no evidence of acute ischemia, hemorrhage, or cerebral edema. She was admitted for work-up of acute stroke and transient ischemic attack and treatment of her hypertensive emergency. Her aphasia resolved within 24 hours after onset, and a modest reduction in BP was achieved. Shortly after admission, however, she developed acute onset of dyspnea, and rales were heard bilaterally during her physical examination.

**Figure 1:**
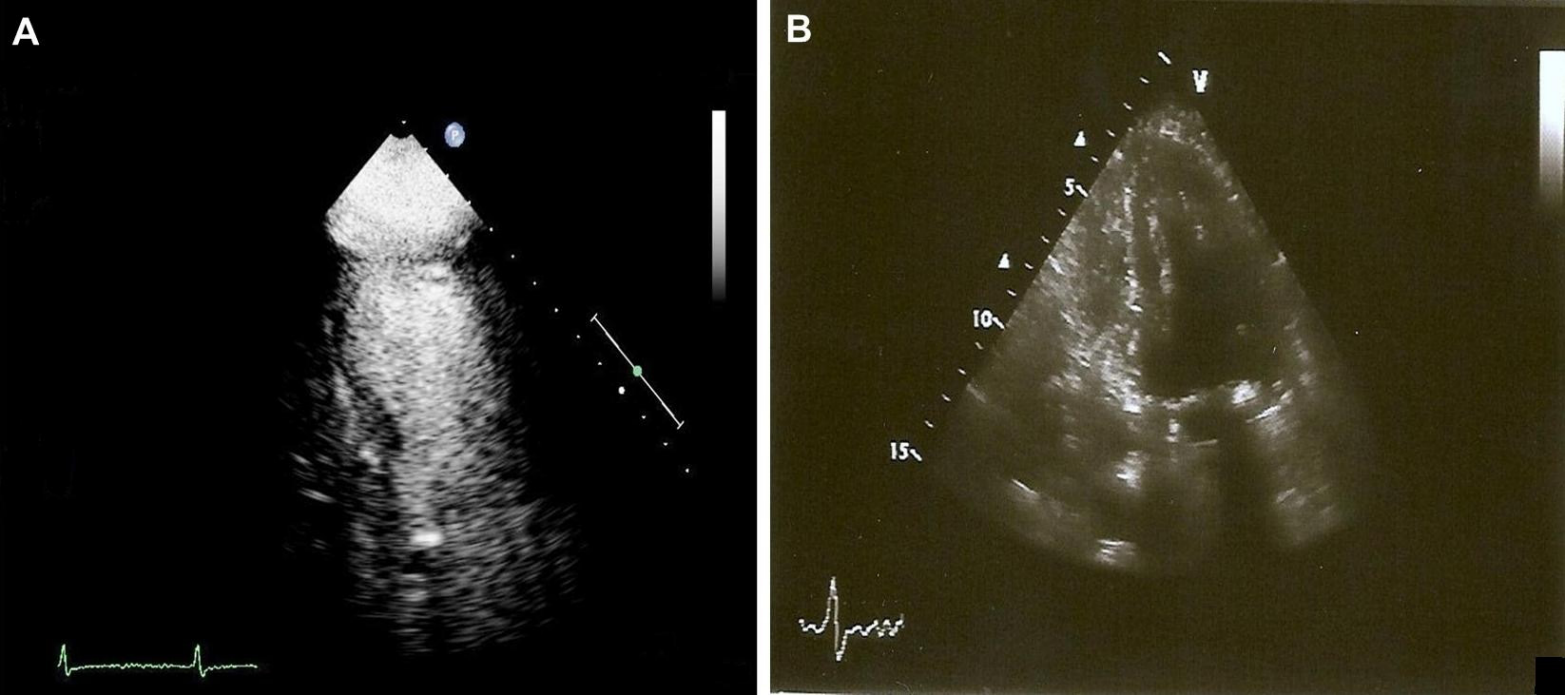
**Echocardiogram on initial presentation and follow-up.** (A) Echocardiogram with contrast enhancement revealing apical ballooning during the patient's first presentation to our hospital. (B) Echocardiogram revealing normally contracting apex with complete recovery of ejection fraction one month after the patient's initial presentation.

In light of these new symptoms, a chest X-ray was ordered, which revealed bilateral pulmonary vascular congestion. Her electrocardiogram (ECG) was unremarkable for ST elevation or depression, though she was found to have elevated cardiac enzymes on serial measurements (troponin I levels 0.8 ug/L and 0.90 ug/L, respectively). An echocardiogram obtained at that time revealed apical akinesis and depressed LV systolic function, with a LVEF of 25% and a possible apical thrombus (Figure 1A). Cardiac catheterization was planned to assess whether the patient had acute CHF secondary to possible acute myocardial infarction. The patient and her family refused to allow us to perform the procedure, however, as she had had a normal cardiac catheterization two years earlier.

Given the clinical, laboratory, and echocardiography data, the patient was started on anti-platelet and anti-coagulation therapy and optimized on heart failure treatment for presumed CHF secondary to ischemic cardiomyopathy. Her symptoms improved, and she was discharged from the hospital with follow-up planned in the out-patient clinic. At one month post-presentation, an echocardiogram revealed complete improvement of LV function back to baseline level (LVEF of 55%) and no evidence of wall motion abnormalities or thrombus (Figure 1B).

**Figure 2:**
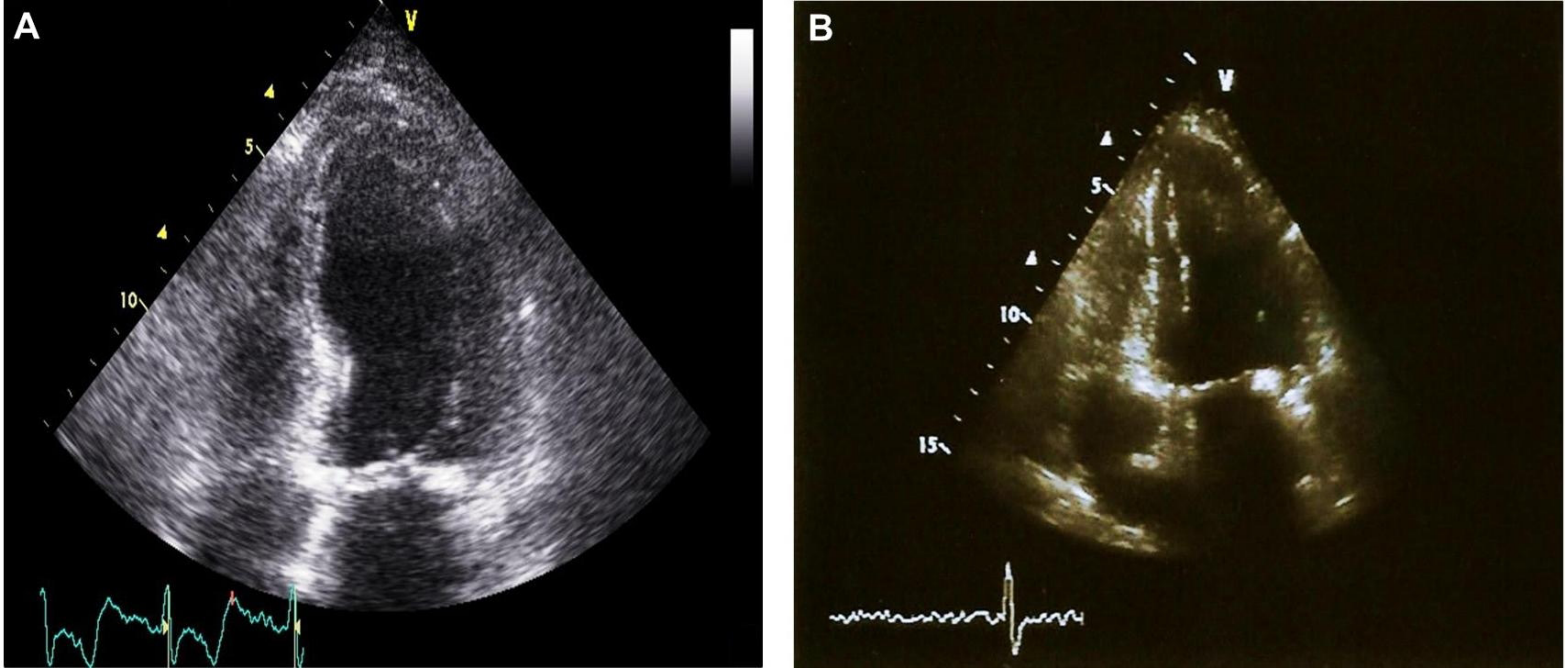
**Echocardiogram on second presentation and follow-up.** (A) Echocardiogram revealing akinesis of the apex during the patient's second presentation to our hospital. (B) Echocardiogram showing normally contracting apex with complete recovery of ejection fraction after the patient's second presentation.

Seven months after her initial presentation, the patient returned to the emergency room for ataxia, dizziness, and associated nausea without focal neurological symptoms. MRI/MRA of her brain was inconclusive, and clopidogrel was added to her treatment regimen based on the suspicion of vertebrobasilar insufficiency. At the time of presentation, an ECG revealed new T-wave inversions in the anterolateral leads and QT prolongation (QTc 490 milliseconds), along with elevated levels of troponin I on serial measurements (0.50 ug/L, 0.89 ug/L, and 0.91 ug/L, respectively) Additionally, during this hospital stay, she again developed symptoms of CHF with worsening shortness of breath, orthopnea, and pedal edema. Her echocardiogram showed a LVEF of 15% to 20% with apical and septal akinesis but without evidence of thrombus (Figure 2A). The patient was treated for CHF and was stable with resolution of her symptoms at the time of discharge. As before, an echocardiogram obtained at her one-month follow-up examination showed complete reversal of her LV systolic function to her normal baseline without evidence of any wall motion abnormality (Figure 2B).

## Discussion

Our patient presented twice with various neurological complaints, and on each occasion she was found to have evidence of acute onset severe apical LV dysfunction that completely resolved within one month of onset. After her first presentation, we suspected that this patient had developed TTC. This condition is a syndrome of transient diminished LV apical wall motion with relative preservation of the basal heart segment in the presence of normal coronary arteries [2]. First described in Japan [1], TTC has since been identified worldwide [8]. Named for the ventricle's similar appearance on ventriculograms to a takotsubo, or Japanese octopus trap, this syndrome, also known as left apical ballooning syndrome or "broken heart syndrome," has been linked to emotional or physical stress, with a high incidence among post-menopausal women. As the syndrome is transient, LV function typically normalizes within six to eight weeks of onset [2].

Our patient is unique in that she had a recurrence of this syndrome within seven months of her initial presentation, and both episodes were preceded by transient neurological complaints. Recurrence of TTC, once thought to be rare, has been increasingly recognized in the literature, with a recurrence rate of 5% to 10% worldwide [2,4,5]. Additionally, the development of TTC in the setting of fleeting neurological symptoms such as aphasia and ataxia without structural brain disease has never been reported. Histological and nuclear imaging data in humans have shown regional differences in efferent sympathetic innervation where the basal ventricular wall possesses a greater mean density of nerve endings and local catecholamine concentrations compared to the apex, while the apex possesses higher concentrations of adrenoreceptors. This differential distribution is proposed to induce the LV dysfunction found in patients with TTC [2,9,10].

Similarly, neurogenic myocardial stunning has also been proposed as a cause of the findings in patients with TTC [2,11]. LV dysfunction with a takotsubo-like state is often seen with intracranial pathology, including subarachnoid hemorrhage and congenital brain abnormalities in children. In the setting of intracranial injury or dysfunction, such as the catecholamine toxicity described in our patient, a surge in sympathetic release will cause a relative hyperdynamic contraction of the LV basal segments with a relative stunning, or ballooning, of the LV apical portion due to saturation of the adrenoreceptors in that distribution [2].

Therefore, there appears to be a correlation between intracranial and LV dysfunction. In fact, recurrence of TTC in association with underlying chronic neurological pathology, mainly status epilepticus, has previously been reported [6,7]. Our present report, however, is the first to describe a patient with recurrent TTC in the setting of transient neurological symptoms without structural evidence of neurologic dysfunction.

## Conclusions

Although no clearly defined etiology for TTC exists, clinicians should be aware of the possibility of TTC in patients whose presentation mimics acute myocardial infarction, especially in the setting of emotional, physical, and specifically neurological stress. Additionally, it has been reported that patients with one episode of TTC are at increased risk for recurrence [2]. As our patient's two presentations suggest, the development and recurrence of TTC likely involve a neurocardiogenic mechanism. Though this condition rarely leads to death, it is imperative that the clinician be aware of this syndrome to ensure the prompt initiation of appropriate supportive care so that a return of normal LV function can be achieved [2]

## Consent

Written informed consent was obtained from the patient for publication of this case report and any accompanying images. A copy of the written consent is available for review by the Editor-in-Chief of this journal.

## Competing interests

The authors declare that they have no competing interests.

## Authors' contributions

The patient was admitted to our hospital under the care of MRS, CK, and TS. JAM, NHA, and WS were major contributors to the researching, writing, and editing of the manuscript. All authors read and approved the final manuscript.
